# Gene based message passing for drug repurposing

**DOI:** 10.1016/j.isci.2023.107663

**Published:** 2023-08-18

**Authors:** Yuxing Wang, Zhiyang Li, Jiahua Rao, Yuedong Yang, Zhiming Dai

**Affiliations:** 1 School of Computer Science and Engineering, Sun Yat-sen University, Guangzhou 510000, China

**Keywords:** Complex system biology, Neural networks, Pharmacoinformatics, Biological constraints

## Abstract

The medicinal effect of a drug acts through a series of genes, and the pathological mechanism of a disease is also related to genes with certain biological functions. However, the complex information between drug or disease and a series of genes is neglected by traditional message passing methods. In this study, we proposed a new framework using two different strategies for gene-drug/disease and drug-disease networks, respectively. We employ long short-term memory (LSTM) network to extract the flow of message from series of genes (gene path) to drug/disease. Incorporating the resulting information of gene paths into drug-disease network, we utilize graph convolutional network (GCN) to predict drug-disease associations. Experimental results showed that our method GeneDR (gene-based drug repurposing) makes better use of the information in gene paths, and performs better in predicting drug-disease associations.

## Highlights

• Gene path information is often neglected in traditional message passing methods
• We proposed GeneDR to perform message passing along gene paths to drugs or diseases
• Gene path information contributes to link prediction between drug and disease

## Graphical abstract

**Figure:**
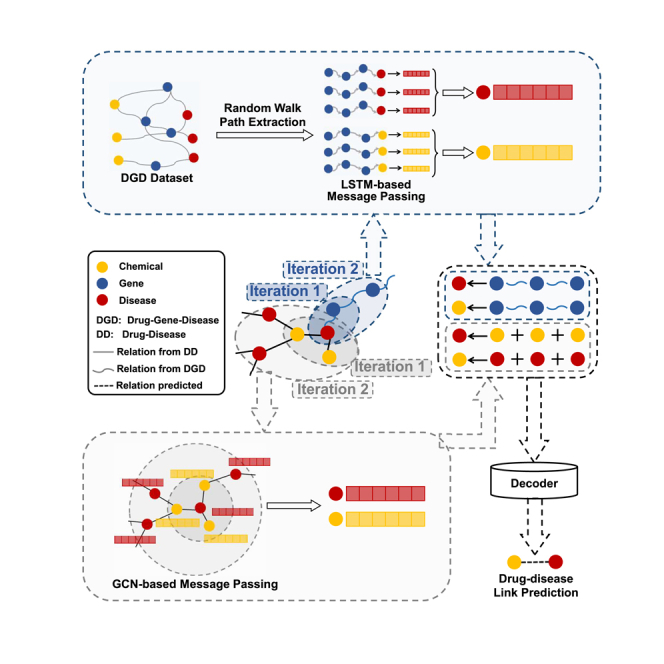

## Introduction

Drug discovery is time-consuming, costly, and laborious. Discovering a new drug normally takes 13–15 years and costs more than a billion dollars on average from development to clinical use.^1^ Computational methods to identify drug-disease associations have attracted increasing attention in the pharmaceutical industry. *In silico* drug repurposing can identify new indications for existing approved drugs and suggest drug candidates for wet lab validation. Drug repurposing can narrow down the search space for the existing drugs and is thus an efficient and promising strategy for traditional drug discovery and development.

As deep learning developed rapidly, neural networks have been applied to drug repurposing, which is to predict the relation between drug and disease. Initially, feature based methods were widely used, which focus on feature extraction by combining multiple biological data related to drug or disease, such as DeepDR.^2^ These data can be constructed as a complex network. Feature extraction methods generally translate the data to vector representations, whereas the topology of network is usually neglected. Graph neural network (GNN) is frequently applied to predict drug-disease relation over recent years, in which a drug or one disease is modeled as a node. However, the semantic information between drugs and diseases is rather complicated, and it cannot be entirely represented by a simple two-layer heterogeneous network. Some previous studies incorporated gene information into drug-disease network and applied graph convolutional network (GCN)-based model to perform drug-disease link prediction with moderate success. For instance, Yu et al.^3^ and Coskun et al.^4^ improved GCN-based drug-disease link prediction by incorporating drug-gene and disease-gene relations to calculate embeddings for drugs and diseases. Li et al.^5^ and Meng et al.^6^ introduced the similarity information to enhance link prediction. Long et al.^7^ proposed a Pre-Training Graph Neural Networks based framework named PT-GNN to integrate gene relation data for link prediction in biomedical networks. PT-GNN uses a GCN-based encoder to effectively refine node features by modeling direct dependencies among nodes in the network. Xuan et al.^8^ proposed GFPred, a method based on a graph convolutional auto-encoder and a fully connected auto-encoder with an attention mechanism. GFPred uses a graph convolutional auto-encoder module to calculate topology representations by integrating gene nodes into drug-disease heterogeneous networks.

These GCN-based models adopt the message-passing mechanism to learn node representations that capture both node features and graph topology information. The representation of a node is updated by its direct neighbors in one iteration. As a result, a k-layers GCN model would capture the information of the local graph containing k-hop neighbors of the central nodes. The pharmacological mechanism of a drug or a disease involves a series of gene nodes, which form as gene paths in a heterogeneous graph. The biological functions of the gene path are critical for drug-disease link prediction and also help to interpret prediction results. GCN-based models use multiple layers to aggregate distant node information. However, too many layers may result in limited distinguished information among nodes (i.e., over-smoothing). Some recent studies have made efforts to capture path information. Flam-Shepherd et al.^9^ proposed a graph neural nets using path embedding to learn local substructure of the graph. They concatenated nodes and edges presentations in a path as path embedding. Kawichai et al.^10^ constructed a network based on disease, drug and gene ontology information, and designed meta-path to calculate representations of drug-disease pairs. Zhou et al.^11^ proposed a meta-path-based computational method called NEDD to predict novel associations between drugs and diseases from heterogeneous information, using meta paths of different lengths to explicitly capture direct relationships or high order proximity. Instead of path, subgraph extraction is also a strategy to focus on local topology of nodes. CoSMIG^12^ extracted subgraphs by employing random walk, and improved message passing method by adding edges into nodes updating.

Besides, hypergraph construction is another strategy to capture high-order information. Feng et al.^13^ transformed the graph into hypergraph by designing hyperedge connecting multiple nodes. This structure allows message passing between node sets connected by hyperedges even though these nodes are not directly connected in the graph. Pang et al.^14^ propose a drug-disease association prediction method to extract high-order drug-diseases association information on hypergraph using hypergraph neural network (HGNN). As mentioned above, the pharmacological mechanism of a drug involves series of genes, since the metabolism process of drug is performed by combining with proteins which are gene products. The combined proteins subsequently effect their related proteins through biological processes. In our heterogeneous graph, we simplified them as the edge between gene nodes and drug nodes. The pharmacological mechanism presents as several paths from a drug node to series gene nodes. The same goes for pathological mechanism of disease. Therefore, gene paths represent biological functions of their connected drug or disease, which contributes a lot to drug-disease link prediction. Although some previous works have taken topology information or paths into node updating, it becomes problematic for longer path due to over-smoothing.

**Figure 1:**
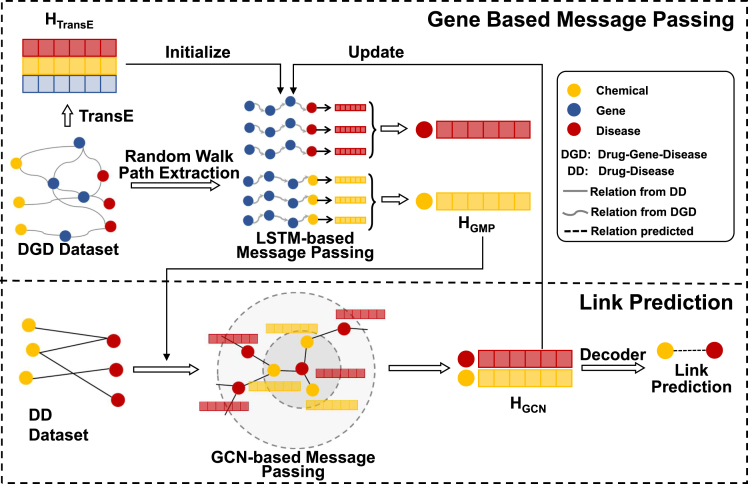
The architecture of GeneDR LSTM-based message passing is performed on extracted gene paths to update the connected drugs and diseases. Subsequently, the updated embeddings are passed on drug-disease bigraph in which GCN-based message passing is used to perform the drug-disease link prediction.

To tackle this, we proposed ac framework, GeneDR (Gene-based Drug Repurposing), to perform message passing along these biological functional series of genes to drug or disease. In our framework, as shown in Figure 1, the gene paths to drug/disease nodes are performed by Long Short-Term Memory (LSTM)-based message passing. LSTM is a special kind of recurrent neural network capable of handling long-term dependencies. Subsequently, the resulting information of gene paths is incorporated into drug-disease network, and GCN based message passing is used to predict drug-disease links. Our framework allows drug/disease nodes to aggregate information along gene paths. Experiment results showed that our method performed better in drug-disease link prediction.

## Results

### Experiment settings

We performed 5-cross validation on two DD datasets, the statistics of which are shown in Table 1. Drug-disease pairs in drug-disease dataset were regarded as positive samples while drug-disease pairs not in drug-disease dataset were randomly chosen as negative samples. The proportion of positive and negative samples is 1:1. The maximal length of gene path was set as 4, and we extracted 100 paths at most for each drug/disease node during one iteration. The hidden size in LSTM and GCN was set as 128, and layer number in GCN was 3. The learning rate was 0.001. All the codes and data are available at github (https://github.com/Wang-yxing/GeneDR).

**Table 1:** The statistics of datasets

Dataset	Drug	Disease	Dr-Di	Gene	Dr-Gene	Di-Gene	Gene-Gene
Dataset 1	268	598	18,416	4,716	65,732	53,474	216,127
Dataset 2	894	454	2,704	31,627	21,634	296,657	1,586,352

Dr, Drug, Di, Disease.

The left part is the original data from Dataset 1 and 2. The right part is corresponded gene data that we collected from PharmKG and CTD.

### Comparison results

We compared our proposed GeneDR with several state-of-the-art methods for link prediction on two datasets. Among them, LAGCN and NIMCGCN are GCN-based methods, which integrate multiple additional data (e.g., entity similarity network) as the node feature. HINGRL utilizes drug structure and disease semantic information as additional features of drug and disease nodes, and calculates the topology feature after performing random walk on the drug-protein-disease heterogeneous graph. DRWBNCF focus on integrating neighborhood interaction of drugs and diseases. It uses localized information in similarity network and drug-disease association network. REDDA collected 5 types of entity and 9 types of networks to construct huge heterogeneous network. It designed topological subnet embedding block to learn node representation. These methods utilize different default data in addition to link prediction data. To optimize the performance for these methods, we used their default data in our experiment. Note that the comparison was based on the same drug-disease association. As shown in the Table 2, GeneDR performed the best. The result indicates that GeneDR makes better use of gene information.

**Table 2:** Comparison results for different methods

Method	AUPR	AUC	F1_score	Recall
**Dataset 1**				

NIMCGCN^5^	0.668	0.181	0.26	0.197
HINGRL^18^	0.918	0.241	0.283	0.248
LAGCN^3^	0.809	0.247	0.223	0.356
DRWBNCF^6^	0.79	0.352	0.416	0.347
REDDA^19^	0.922	0.444	0.495	0.451
GeneDR	0.935	0.464	0.501	0.476

**Dataset 2**				

NIMCGCN^5^	0.675	0.238	0.295	0.43
HINGRL^18^	0.809	0.401	0.439	0.529
LAGCN^3^	0.848	0.521	0.506	0.564
DRWBNCF^6^	0.848	0.477	0.490	0.555
REDDA^19^	0.869	0.548	0.528	0.565
GeneDR	0.883	0.579	0.554	0.583

### Ablation study

**Figure 2:**
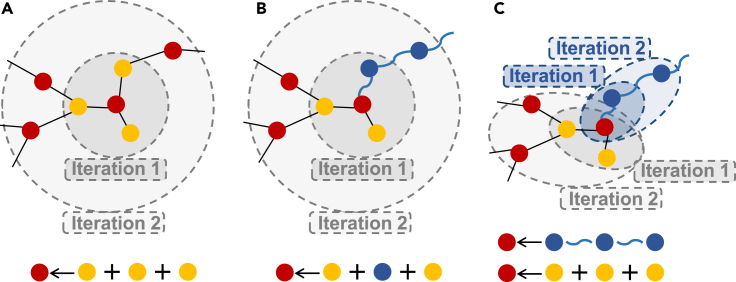
Comparison of message passing methods The yellow, red and blue nodes rep-resent drug, disease and gene, respectively. For convenience, we take disease as central node for illustration. The black straight lines represent relations between drugs and diseases, while the blue curly lines represent relations between genes and diseases. (A) The traditional message passing on drug-disease bigraph where the node embedding is updated by the surrounded homogeneous nodes. (B) The traditional message passing on drug-gene-disease heterogeneous graph where the node embedding is updated by the surrounded heterogeneous nodes. (C) Message passing separately on drug-disease bigraph (gray) and on gene path (blue).

We also conducted ablation studies to investigate factors that influence our performance as shown in Table 3. We designed two variants of GeneDR: GeneDR without GMP (w/o GMP) performs message passing as in Figure 2B; GeneDR without LSTM (w/o LSTM) uses GCN to aggregate gene message along the path instead of LSTM. GeneDR w/o LSTM performed better than GeneDR w/o GMP, suggesting that separating message passing of genes to drugs or diseases from message passing between drugs and diseases contributes to drug-disease link prediction. The two variants were inferior to GeneDR, indicating that LSTM-based message passing makes better use of gene path information probably by simulating flow of message along the gene path.

**Table 3:** Ablation experiment results

Method	AUPR	AUC	F1_score	Recall
GeneDR w/o GMP^a^	0.8431	0.472	0.480	0.552
GeneDR w/o LSTM^b^	0.856	0.503	0.505	0.562
GeneDR	0.883	0.579	0.554	0.583

a Link prediction on combination of DD dataset and DGD dataset without the gene path extraction (GPE).

b Gene path message passing on GCN instead of LSTM.

### Case study

To demonstrate the practical ability of GeneDR for identifying drug-disease interactions, we conducted case studies by literature evidences (see Table 4 for some examples, the full list of predicted drug-disease interactions and the related gene paths was provided in GitHub). Interestingly, we found some predicted drug-disease links represent no therapy but side effect. For instance, prediction results showed that asthma is highly related to indomethacin, diclofenac, and nicotine, which were reported to lead to asthma.^15^^,^^16^^,^^17^ These results suggest that our framework can predict the related drugs and diseases, but cannot distinguish between the therapeutic relation and side effect relation, which motivate us to take the up- or downregulation between genes in gene path into consideration in further work.

**Table 4:** Same examples of the drug-disease prediction results and literature evidences

Drug	Disease	Evidence
Carbamazepine	Chorea	Genel et al.^20^; Harel et al.^21^
Furosemide	Asthma	Pendino et al.^22^; Inokuchi et al.^23^
Docetaxel	Colorectal Neoplasms	O’Brien et al.^24^; Guo et al.^25^
Risperidone	Epilepsy	Holzhausen et al.^26^; Mula et al.^27^; Penagarikano et al.^28^
Olanzapine	Epilepsy	Qiu et al.^29^
Methotrexate	Myocarditis	Campochiaro et al.^30^; Li et al.^31^
Indomethacin	Peritonitis	Peng et al.^32^
Tretinoin	Urinary Bladder Neoplasms	Laaksovirta et al.^33^; Polat et al.^34^

### Conclusion

We propose a new framework to perform message passing along the gene paths to their connected drugs or diseases. Thus, the gene information of paths is aggregated to update the embeddings of the drugs and diseases, which is demonstrated to contribute to the link prediction between drug and disease. Furthermore, we believe that our identified gene paths of the drug and disease will be useful to explain the predicted drug-disease link.

### Limitations of the study

As mentioned in Results, we did not introduce relation type between genes in gene paths. Relation types, such as upregulation and downregulation, are very important information when distinguishing the specific relation between drug and disease. For example, a disease and a drug are probably related when they are associated to same genes, but the up- or downregulations between them and genes determine whether the disease is treated by the drug or is a side effect of the drug. In our project, we only focus on whether there is relation between drug and disease instead of the type of the relation. It is worth considering gene relation type in our future work.

## STAR★Methods

### Key resources table

**Table:** 

REAGENT or RESOURCE	SOURCE	IDENTIFIER
**Deposited data**		

Dataset 1	Yu et al.^3^	https://github.com/storyandwine/LAGCN
Dataset 2	Gu et al.^19^	https://github.com/gu-yaowen/REDDA
PharmKG	Zheng et al.^37^	https://github.com/MindRank-Biotech/PharmKG
Comparative Toxicogenomics Database (CTD)	Davis et al.^36^	http://ctdbase.org/
Initial node embeddings	This paper	https://github.com/Wang-yxing/GeneDR
Gene path	This paper	https://github.com/Wang-yxing/GeneDR

**Software and algorithms**		

Python	Python Software Foundation	https://www.python.org
Pytorch	Pytorch Software Foundation	https://pytorch.org/
TransE	Borders et al.^35^	https://github.com/thunlp/OpenKE
GeneDR	This paper	https://github.com/Wang-yxing/GeneDR

### Resource availability

#### Lead contact

Further information should be directed to and will be fulfilled by the lead contact, Prof. Zhiming Dai (daizhim@mail.sysu.edu.cn).

#### Materials availability

This study did not generate new unique materials.

### Method details

#### Data overview and data preprocessing

##### Drug-disease pair

To be consistent with drug-disease association dataset used by other methods, we chose two drug-disease datasets. Dataset 1^3^ contains 268 drugs, 598 diseases and 18416 relations between diseases and drugs. Dataset 2^19^ contains 894 drugs, 454 diseases and 2704 relations between diseases and drugs.

##### Gene-gene pair

To extract gene path, gene-gene relations are collected from our constructed database PharmKG,^35^ which integrates multi-omics data with more than 500,000 relations between genes, drugs and diseases. Restricted from gene path length, the genes that are multi-hops away from any disease or drug node are filtered out in this work.

##### Disease/drug-gene pair

Disease-gene and drug-gene pairs are also obtained from PharmKG. Note that we only keep the targeted drugs and diseases in Dataset 1 or 2. Besides, the drug and disease that not in PharmKG is completed by CTD,^36^ which also provides drug or disease related genes collected from existed experiments and auto literature curation. We only keep the pairs from experiments to assure the data quality. The detailed statistics is shown in Table 1. The left part is drug-disease datasets (DD datasets), and right part is drug-gene-disease datasets (DGD datasets) we constructed according the diseases and drugs in DD datasets.

##### Initial node embedding

The initial node embeddings are obtained by training TransE^37^ on DGD datasets separately. TransE is a translation based model, which represents relations as translations in the embedding space. The basic idea of TransE is to learn entity and relation embeddings in triple with the condition that head entity embedding plus relation embedding approximately equals to tail embedding. Therefore, it can integrate global information for every node in DGD dataset.

##### Gene path

Gene paths for each drug and disease are extracted from DGD dataset by Random Walk.^38^ In each path, the start node is drug or disease and subsequent nodes are genes. The length of paths is set as 4, and we extracted 100 paths at most for each drug/disease.

#### Problem definition

In a graph G=(V,E), V is the set of nodes containing gene $V_{g}$, disease $V_{d}$ and drug $V_{r}$, while E is the set of edges among nodes. P denotes the entire set of gene paths, and $P_{i}$ denotes set of gene paths started with a disease or drug node *i* followed by a series of gene nodes, where $P_{i}\subset P$ and $i\in \left\{V_{d},V_{r}\right\}$.

#### Traditional message passing

In traditional message passing method, node embedding is updated by the directly connected neighbors during each iteration:

(Equation 1) $$m_{i}^{\left(l\right)}=aggregate^{\left(l\right)}\left(\left\{h_{j}^{\left(l-1\right)}:j\in N_{i}\right\}\right)\text{,}$$

(Equation 2) $$h_{i}^{\left(l\right)}=update\left(\left\{h_{i}^{\left(l-1\right)},m_{i}^{\left(l\right)}\right\}\right)\text{,}$$

where $h_{i}^{\left(l\right)}$ is the embedding of the node *i* in *l*-th layer, $N_{i}$ is the direct neighbors of node *i*, $h_{j}$ is the embedding of the direct neighbors. $m_{i}^{\left(l\right)}$ is the message aggregated from the neighbors, which is used to update the node embedding.

Figures 2A and 2B shows node embedding in traditional message passing under two circumstances. Figure 2A illustrates the embedding of a drug/disease node is updated by the surrounded drug/disease nodes during alternate iterations in a drug-disease bigraph. Take the central disease node in Figure 2A as example, the information of the surrounded drug nodes is aggregated into the central disease node embedding in the first iteration, and in the next iteration, message passing will spread out to the further nodes. The aggregated nodes are homogeneous at each iteration in the bigraph, which is in accord with the mechanism of traditional message passing method. However, the message passing process becomes problematic when gene nodes are added into the graph. As shown in Figure 2B, the central disease node is surrounded by genes and drugs. When using traditional message passing methods, the messages from gene nodes and drug nodes are aggregated together at one iteration. Besides, the gene nodes in a path are separated by several iterations without making full use of their information.

#### Gene based path message passing

Taken gene path into consideration, we revised the message passing method (Figure 2C). Our proposed message passing framework contains two parts, one is gene based message passing which integrated node information along gene paths, the other is drug-disease message passing, which is the same as Figure 2A. Gene messages are aggregated as below:

(Equation 3) $$m_{i}^{\left(l\right)}=\Sigma_{P_{i}}a_{k}LSTM^{\left(l\right)}\left(\left\{p_{k},H^{\left(l-1\right)}:p_{k}\in P_{i}\right\}\right)\text{,}$$

(Equation 4) $$h_{i}^{\left(l\right)}=update\left(\left\{h_{i}^{\left(l-1\right)},m_{i}^{\left(l\right)}\right\}\right)\text{,}$$

where $m_{i}^{\left(l\right)}$ is the message aggregated from the set of the paths $P_{i}$ connected with node *i*, and $a_{k}$ is the trainable weight of the path $p_{k}$ among the paths in $P_{i}$, $p_{k}\in P_{i}$.

$H^{\left(l-1\right)}$ is the node embedding matrix from last layer. We employ LSTM to perform message passing along the path. The hidden state of the terminal node in the path is regarded as the message vector aggregating all information of this path. In the path from genes to disease or drug, the hidden state of the drug or disease node can capture the information of all genes in the path. Since each drug or disease is generally connected with more than one path, we introduce path weight acted as attention mechanism to integrate the connected paths and to distinguish their respective importance.

#### The architecture of GeneDR

As shown in Algorithm 1 and Figure 1, the initial node embeddings, $H_{TransE}$, are obtained by training TransE on DGD dataset, which can integrate global information for every node. Gene paths for each drug and disease are also extracted from DGD dataset by Random Walk. Gene based message passing (GMP) is then performed along paths to the connected drug and disease nodes through LSTM. The resulting embeddings, $H_{GMP}$, are used to initialize drug and disease nodes in the drug-disease bigraph.

Algorithm 1 **Overview of GeneDR**

**Input:** DD dataset $G_{1}=\left(V_{1},E_{1}\right)$, $V_{1}=\left\{V_{d},V_{r}\right\}$;
  DGD dataset $G_{2}=\left(V_{2},E_{2}\right)$, $V_{2}=\left\{V_{d},V_{r},V_{g}\right\}$.
**Output:** Drug-disease link prediction value v(r,d) between drug $r\in V_{r}$ and disease $d\in V_{d}$. Calculate the node embedding $H_{\text{TransE}}$ from $G_{2}$ by using TransE. Extract gene paths *P* for each node in $V_{1}$ by performing random walk on $G_{2}$.
**for** each epoch **do**
  **for** round = 2 **do**
    $H_{\text{GPEMP}}\leftarrow GMP\left(H_{\text{TransE}},P\right)$ with Equation 4.
    $H_{\text{GCN}}\leftarrow GCN\left(H_{\text{GPEMP}},G_{1}\right)$ with Equation 2.
  **end**
  **for** each link (r,d) **do**
    $v\left(r,d\right)\leftarrow predictor\left(H_{GCN}^{2}\right)$
  **end**
**end**

The information in drug-disease bigraph is aggregated by GCN-based message passing and is output as $H_{GCN}^{1}$. To better use the information, i.e., gene path and drug-disease bigraph, $H_{GCN}$ is back to LSTM-based layer to update around the workflow again. Eventually, after two round, drug and disease embeddings from final $H_{GCN}$ are concatenated and input into a fully connected layer to output the final link prediction.

### Quantification and statistical analysis

We performed 5-cross validation on two DD datasets. Drug-disease pairs in drug-disease dataset were regarded as positive samples while drug-disease pairs not in drug-disease dataset were randomly chosen as negative samples. The proportion of positive and negative samples is 1:1. We assessed model performance by using common metrics including: AUPR (area under the precision-recall curve), AUC (area under the curve) score, F1 score, Recall.

## Data Availability

• The data mentioned in this paper are publicly available, and are listed in key resources table with accessibility. • All the codes are available online at Github and is fully publicly available as of the date of publication. • Any additional information required to reanalyze the data reported in this paper is available from the lead contact upon request.
